# PUM2 aggravates the neuroinflammation and brain damage induced by ischemia–reperfusion through the SLC7A11-dependent inhibition of ferroptosis via suppressing the SIRT1

**DOI:** 10.1007/s11010-022-04534-w

**Published:** 2022-08-23

**Authors:** Qingran Liu, Yongchang Liu, Yan Li, Zhen Hong, Shaoquan Li, Chen Liu

**Affiliations:** 1 Department of Neurovascular Intervention, Cangzhou Central Hospital, No. 16, Xinhua West Road, Cangzhou, 061000 Hebei China; 2 Department of Neurosurgery, Cangzhou Central Hospital, No.16, Xinhua West Road, Hebei 061000 Cangzhou, China

**Keywords:** PUM2, SIRT1, SLC7A11, Neuroinflammation, Brain damage, Ischemia–reperfusion

## Abstract

Cerebral ischemia–reperfusion (I/R) injury occurs due to the restoration of blood perfusion after cerebral ischemia, which results in the damage of the brain structures and functions. Unfortunately, currently there are no effective methods for preventing and treating it. The pumilio 2 (PUM2) is a type of RBPs that has been reported to participate in the progression of several diseases. Ferroptosis is reported to be involved in I/R injury. Whether PUM2 modulated I/R injury through regulating ferroptosis remains to be elucidated. The cerebral I/R models including animal middle cerebral artery occlusion/reperfusion (MCAO/R) model and oxygen–glucose deprivation/reperfusion (OGD/R)-induced cortical neuron injury cell model of were established and, respectively. RT-qPCR was applied for evaluating PUM2, SIRT1 and SLC7A11 expression. Western blot was employed for measuring the protein expression levels. The viability of cortical neurons was tested by MTT assay. The histological damage of the brain tissues was assessed by H&E staining. The level of PUM2 was boosted in both the brain tissues of the MCAO model and OGD/R-induced cortical neuron injury model. Silence of PUM2 alleviated MCAO-induced brain injury and decreased the death of PC12 cell exposed to OGD/R. PUM2 also aggravated the accumulation of free iron in MCAO mice and OGD/R-induced cortical neuron injury model. In addition, PUM2 suppressed SLC7A11 via inhibiting expression of SIRT1. Rescue assays unveiled that downregulation of SLC7A11 reversed PUM2 mediated neuroinflammation and brain damage induced by I/R. PUM2 aggravated I/R-induced neuroinflammation and brain damage through the SLC7A11-dependent inhibition of ferroptosis by suppressing SIRT1, highlighting the role of PUM2 in preventing or treating cerebral I/R injury.

## Introduction

Stroke is one of the cerebral blood circulation disorders associated with a major cause of long-term neurological disability and death worldwide [1]. It is estimated that approximately 1/3 of all deaths were resulted from stroke all over the world [2]. About 75–85% of the stroke cases were ischemic stroke [3]. Ischemic stroke refers to the blood flow obstruction to brain due to the occlusion of the major cerebral aorta [4]. Rapid restoration of the blood supply to the brain was the required intervention for the treatment of ischemic stroke [5]. Reperfusion of ischemic brain tissues is a method for restoring the blood supply to the brain, which prevents severe neurological impairment and death, but it can also lead to cerebral ischemia–reperfusion (I/R) injury resulting in nerve damage [6]. To date, the detailed pathogenesis and mechanisms of the cerebral I/R injury remains unclear. Novel biomarkers were required to elucidate the pathogenesis for preventing the occurrence of cerebral I/R injury.

Ribonucleic acid binding proteins (RBPs) are for post-transcriptionally modulating the mRNA stability and translation to control the levels of proteins expression [7]. The pumilio (PUM) family is a type of RBPs that mediates the expression of mRNAs via targeting their 3′-untranslated regions (3′-UTRs) [8]. A complex is formed in PUM and Nanos to bind with specific mRNAs and recognize the PUM-binding elements (PBE) in the 3′-UTRs of target mRNAs for regulating the degradation of mRNAs [9]. PUM2 is a member of the PUM family, which has been reported to participate in the progression of several diseases. For instance, PUM2 modulates the resistance to cisplatin in ovarian cancer via regulating USP46 expression [10]. PUM2 governs mitochondrial quality in acute ischemic kidney injury through cooperating with Mff [11]. Moreover, PUM2 is validated to facilitate the development of mature neurons [12].

Interestingly, it was discovered that PUM2 can reduce the stability of SIRT1, thus down-regulating its expression [13]. Sirtuin 1 (SIRT1) is low expressed, and the higher expression of SIRT1 protects brain tissues from I/R injury by restoring mitochondrial homeostasis, regulating oxidative stress and inflammatory response, and inhibiting neuronal apoptosis [14–16]. SIRT1 is frequently reported to be involved in cerebral I/R injury. For example, lncRNA MALAT1/miR-142-3p/SIRT1 axis modulates cerebral I/R injury and cognitive dysfunction [17]. SIRT1 is targeted by miR-7-5p to mediate the cerebral I/R injury [18]. The neuroprotective role of SIRT1 is to protect neural function from cerebral I/R via modulating PGC-1α signaling [19]. However, whether PUM2 is involved in the progression of cerebral I/R injury through regulating SIRT1 remains unclear.

Herein, this study was aimed to explore the role of PUM2 in cerebral I/R injury. The results depicted that PUM2 aggravated the I/R-induced neuroinflammation and brain damage through the SLC7A11-dependent inhibition of ferroptosis via suppressing the SIRT1.

## Methods

### Cell culture and oxygen–glucose deprivation/reperfusion (OGD/R) treatment

The primary mouse cerebral cortical neurons were separated from C57BL/6 mice, as previously reported [20]. After being incubated in Dulbecco’s modified Eagle’s medium (DMEM) with glucose (4.5 g/mL), heat-inactivated fetal bovine serum and horse serum (5%, Gibco), cortical neurons were rinsed by phosphate buffer saline (PBS). Then, cortical neurons were grown in Earle’s balanced salt solution containing CaCl_2_ (0.9 mM), KCl (5.4 mM), NaH_2_PO_4_ (l mM), MgSO (40.8 mM), NaCl (116 Mm), and 10 mg/L phenol red (10 mg/L) followed by transferring to a chamber containing oxygen (1%), CO_2_ (5%) and N_2_ (94%). After 2 h, cells were incubated in an indoor condition for 12 h. The cortical neurons cultured in normoxic condition served as the control group [21].

### Lentiviral transduction, plasmid transfection and treatment

Short hairpin RNAs (shRNAs) targeting PUM2 (shPUM2), SIRT1 (shSITR1) and respective shNCs were obtained from GenePharma. Lipofectamine 3000 transfection reagent (Invitrogen) was employed for Lentiviral (LV) transduction or shRNA transfection into the mice or cortical neurons.

### 2,3,5-triphenyltetrazolium chloride (TTC) staining

The infarcted size of the brain tissues was assessed via TTC staining. After being washed with PBS, the brain tissues of the mice were put in the condition of − 20 °C for 10 min and then cut into 2 mm tissue sections. Next, 2% TTC solution (G3005, Solarbio, Beijing, China) (0.2 M phosphate buffer, pH 7.4) was supplemented. After being stained in the dark at 37 °C water bath for 30 min, the tissues sections were fixed by 4% paraformaldehyde (Wako Pure Chemical Industries, Japan) for 2 h and pictures were acquired. The ImageJ software (NIH, USA) was applied for analysis of the images. The white areas indicated the infarcted brain tissues and others referred to the normal tissues (Table 1).

**Table 1:** Basic physiological parameters

	Sham	MCAO
pCO_2_(mmHg)	40.53 ± 4.78	38.99 ± 5.9
pO_2_(mmHg)	110.51 ± 12.04	115.7 ± 21.6
pH(− log[H^+^])	7.4 ± 0.05	7.48 ± 0.03
FBG(mg/dL)	13.92 ± 2.73	71.84 ± 5.72*
MABP(mmHg)	75.67 ± 6.22	112.66 ± 11.1*
ICP(mmHg)	10.96 ± 2.73	31.38 ± 5.78*
CPP(mmHg)	68.62 ± 8.06	95.84 ± 9.99*

(*) P<0.05

### Flow cytometry

Cell apoptosis was explored by flow cytometry. The trypsin was applied for digesting the cortical neurons suspension followed by washing with pre-cooled PBS, and centrifuging for 5 min. Furthermore, 500 μL binding buffer was supplemented following by staining with Annexin V-FITC (5 μL) at indoor temperature for a quarter. Afterward, propidium iodide (PI, 5 μL) was added for staining, and a FC500MCL flow cytometer was applied for detecting the apoptosis.

### Enzyme-linked immunosorbent assay (ELISA)

The concentrations of malondialdehyde (MDA), glutathione (GSH), and glutathione peroxidase (GPX) in the brain tissues of the mice were measured via respective ELISA kits. The optical density (OD) value at 450 nm was evaluated using microplate reader (Thermo Fisher).

### Hematoxylin and eosin (H&E) and terminal deoxynucleotidyl transferase mediated dUTP nick-end labeling (TUNEL) staining

The histological damages of the brain tissues were assessed by H&E staining. Post reperfusion for 24 h, sodium pentobarbital was applied for the anesthetization of the mice followed by perfusion with PBS and 4% paraformaldehyde. Different concentrations of alcohols were used to dehydrate the brain tissues followed by paraffin-embedding. Thereafter, the brain tissues were cut into 5-μm sections and subjected to H&E reagents. The histopathological changes were recorded. Paraffin-embedded brain tissue sections were subjected to TUNEL staining (Roche, Germany). The inverted fluorescence microscope (Leica) was applied to evaluate the images of TUNEL and the rate of apoptotic cells was calculated.

### Western blot

The concentrations of proteins from brain tissues or cortical neurons were evaluated through a bicinchoninic acid protein assay kit (Pierce, Rockford). Then the protein samples (20 μg) were separated by 8% SDS–PAGE followed by transferring onto a polyvinylidene fluoride membrane (Millipore, Billerica, MA). After being blocked by nonfat milk at room temperature for 2 h, the membrane was incubated with primary antibodies at 4 °C overnight. After that, the peroxidase-conjugated goat anti-rabbit IgG (1:2000, Santa Cruz Biotechnology) antibodies were used for incubation for another 1 h. The chemiluminescence (Millipore, Billerica, MA) was applied for the visualization of the protein blots. The primary antibodies were displayed as follows: anti-PUM2 (1:5000, ab92390, Abcam, Shanghai, China), anti-SIRT1 (0.125 µg/mL, ab110304, Abcam), anti-SLC7A11 (1:1000, ab175186, Abcam), anti-acyl-CoA synthetase long-chain family member 4 (ACSL4) (1:10,000, ab155282, Abcam), anti-transferrin receptor 1 (TFR1) (1:1000, ab214039, Abcam), anti-ferritin heavy chain 1 (FTH1) (1:1000, ab183781, Abcam), anti-glutathione peroxidase 4 (GPX4) (1:1000, ab125066, Abcam), and anti-β-actin (1 µg/mL, ab8226, Abcam).

### Middle cerebral artery occlusion/reperfusion (MCAO/R)

Adult male C57BL/6 mice (25–30 g) were obtained from Weitong Lihua Experimental Animal Technology (Beijing, China). The animal experiments got the approval from the Animal Care and Use Committee of Cangzhou Central Hospital. The MCAO/R was performed according to the procedure in previous study [22]. After the anesthetization by isoflurane, the middle cervical skin of the mice was incised followed by isolating the common external carotid artery (ECA), internal carotid artery (ICA), and carotid artery (CCA). A 5-0 nylon monofilament was inserted from CCA into ICA to occlude the MCA for 1 h. In the sham group, the mice received the same procedure except inserting the nylon monofilament.

### 3-[4,5-Dimethylthiazol-2-yl]-2,5 diphenyl tetrazolium bromide (MTT) assay

The viability of cortical neurons was tested via MTT assay. Cortical neurons were grown on 96-well plates at 37 °C with 5% CO_2_ for 0, 24, 48, 72, and 96 h. Further, 10 μL of MTT solution (Sigma, USA) was added for another incubation for 4 h. DMSO was supplemented to stop the reaction. Finally, the absorbance at 570 nm at different time point was evaluated via a microplate reader (Thermo Fisher Scientific).

### The reverse transcription-quantitative polymerase chain reaction (RT-qPCR)

Trizol was applied for extraction of total RNAs followed by reverse transcribing the RNAs into cDNAs via a reverse transcription kit (Takara, Japan). Real-time quantitative PCR was executed on the Roche LightCycler480 (LC480) through the SYBR Premix PCR kit (Takara, Japan). 2^−∆∆Ct^ method was applied for calculating the relative expression levels of PUM2, SIRT1, SLC7A11 with GAPDH serving as the controls. The respective primers were presented as follows:

PUM2

F: 5′-TTCCACAGCCAAGAGACGCA-3′,

R: 5′-GCACTCAGCCACCACAGCAG-3′;

SIRT1

F: 5′-CAAGGGATGGTATTTATGCTCG-3′,

R: 5′-CAAGGCTATGAATTTGTGACAGAG-3′;

SLC7A11

F: 5′-TTGTTTTGCACCCTTTGACA-3′,

R: 5′-AAAGCTGGGATGAACAGTGG-3′;

GAPDH

F: 5′-ATGTTCGTCATGGGTGTGAAC-3′,

R: 5′-ATGGACTGTGGTCATGAGTCC-3′.

### Koeppen’s Perls’ Prussian blue staining

The Koeppen’s Perls’ Prussian blue staining was employed for analyzing the intracellular iron deposition. The paraffin embedded brain tissues were sliced into 10-μm sections followed by hydration with 100%, 95%, 85%, 75% and 70% ethanol for 1 min. Furthermore, the sections were immersed in Perls’ staining solution (80 mL, 20% HCl and 80 mL, 10% potassium ferrocyanide) for 20–30 min. Then the sections were washed by distilled water followed by Eosin staining for 1 min. Afterward, 80%, 85%, 90% and 100% ethanol was filled in the sections followed by hyalinizing the sections with xylene and sealing them with neutral resin. Three fields were applied for each section for calculation.

### Determination of iron content in tissues

Cortical neurons were incubated in iron Assay Buffer on the ice. Post homogenization, cortical neurons were centrifuged at 4 °C at 16,000×*g* for 10 min. The iron content was measured and a microplate reader (Thermo, USA) was applied to examine the absorbance at 593 nm.

### Statistical analysis

The statistical analysis was executed through SPSS19.0 software (SPSS Inc., Chicago, USA). All data were displayed as mean ± standard deviation. Comparisons between or among groups were subjected to *t* test or one-way ANOVA analysis with the Turkey test. All the experiments were repeated for three times and *p* < 0.05 was considered as statistically significant difference.

## Results

### PUM2 expression was boosted in MCAO model and OGD/R-induced cortical neuron injury model

**Fig. 1:**
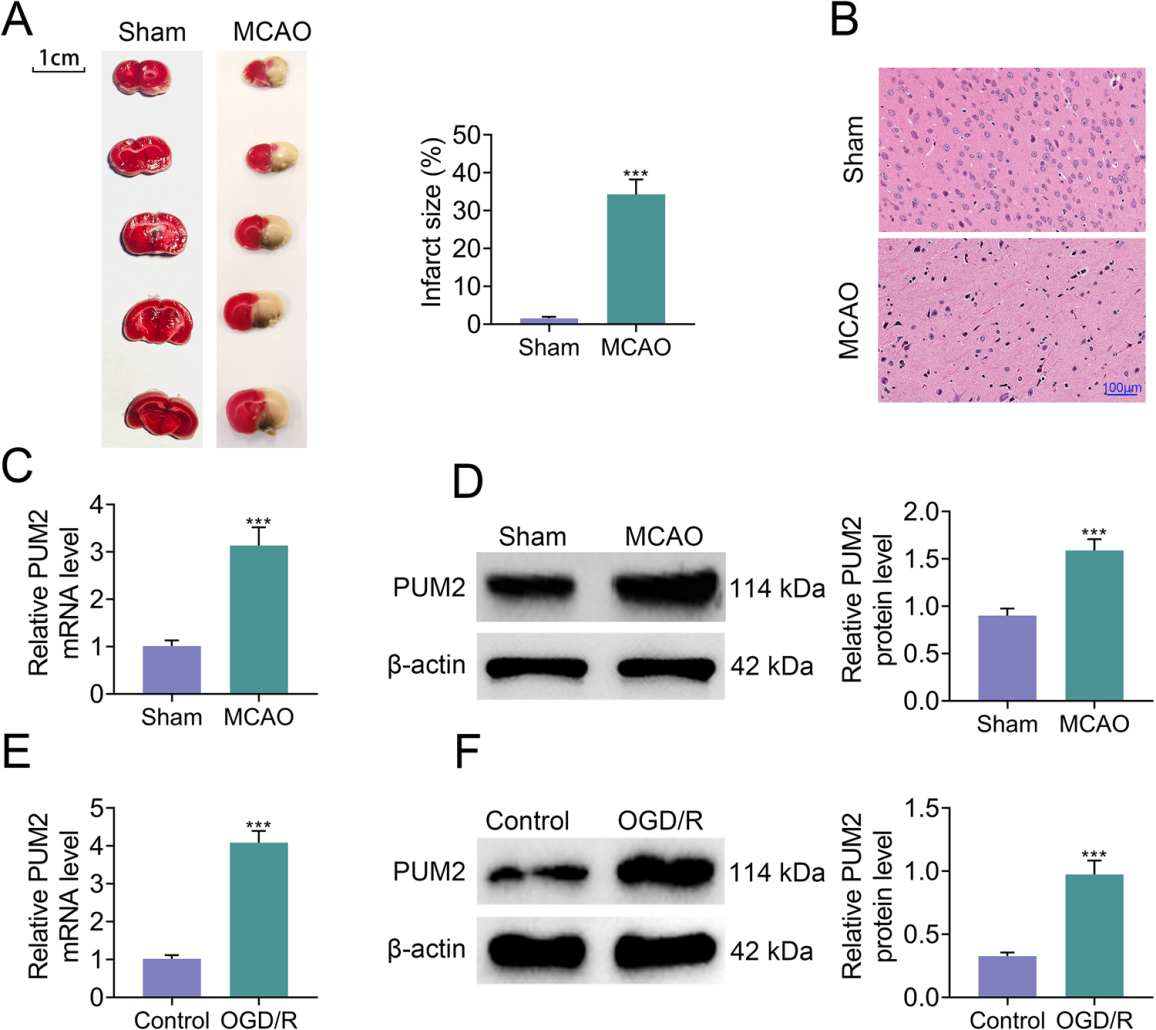
PUM2 expression was boosted in animal model and OGD/R-induced cortical neuron injury model. **A** TTC staining was performed to measure the infarct size of brain tissues. **B** H&E staining revealed the pathological histological damage of the brain tissues. **C**–**F** RT-qPCR and Western blot disclosed the mRNA and protein expression levels of PUM2 in MCAO model and OGD/R model. Five mice were used in each group. ***p < 0.001 compared with sham group or control group

To determine the role of PUM2 in cerebral I/R, the animal and cell models of cerebral I/R were established, respectively. As exhibited in Fig. 1A, the infarcted size of the brain tissues in the MCAO group was evidently larger than the sham group. H&E staining also revealed severer edema and nuclear division in the nerve cells and more vacuoles and edema in the tissue space were observed in MCAO group compared to sham group (Fig. 1B). The damaged brain tissues in the MCAO group indicated that the animal model was successfully established. Thereafter, the mRNA and protein levels of PUM2 were evaluated in the brain tissues, and the results showed that mRNA and protein expression of PUM2 was strikingly elevated in the brain tissues of MCAO group compared to the sham group (Fig. 1C, D). In addition, the mRNA and protein expression of PUM2 was also markedly increased in the OGD/R group in comparison with the control group (Fig. 1E, F). Altogether, these results indicated that PUM2 expression was boosted in MCAO model and OGD/R-induced cortical neuron injury model.

### PUM2 restraint alleviated MCAO-induced brain injury

**Fig. 2:**
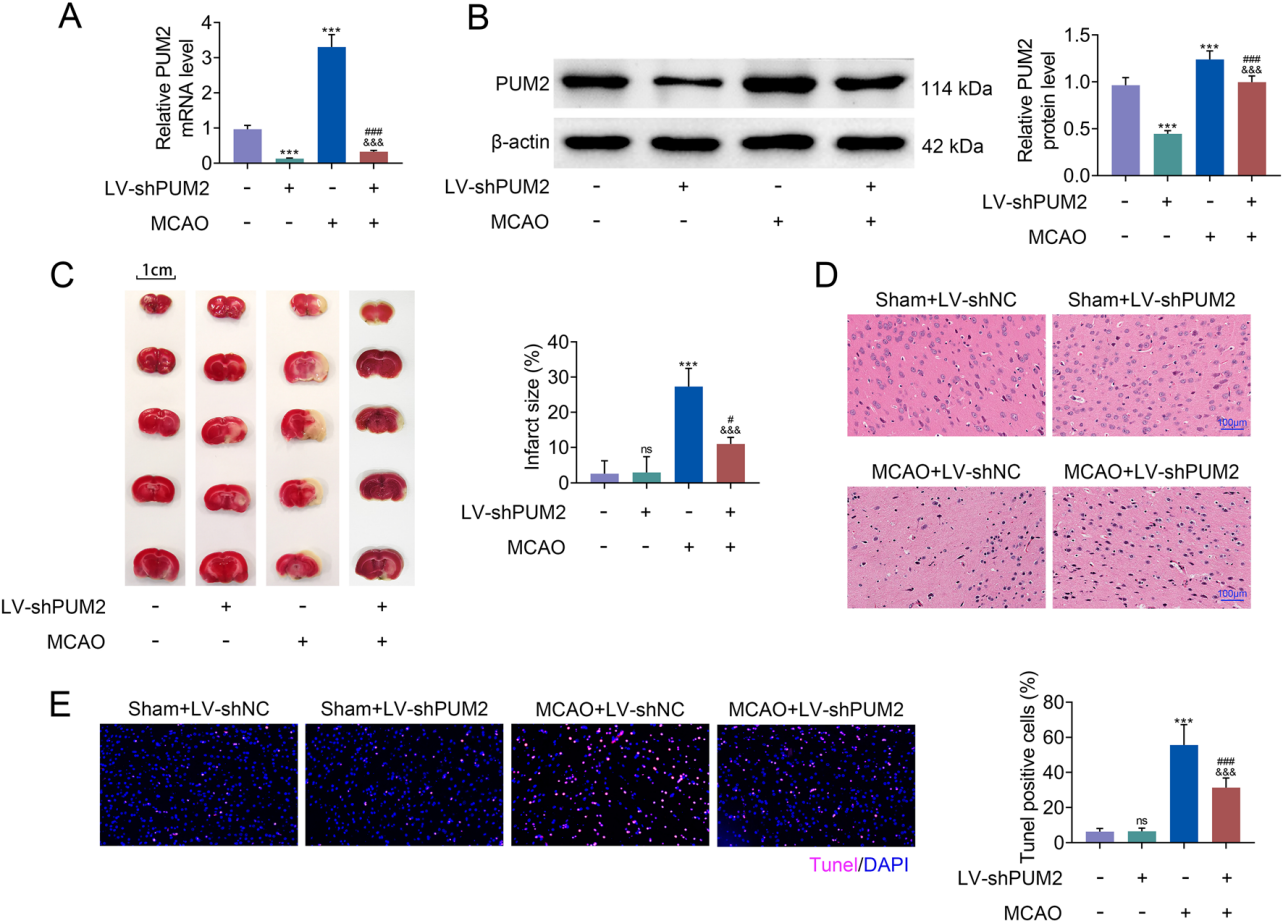
PUM2 restraint alleviated MCAO-induced brain injury. **A** The knockdown efficiency of LV-shPUM2 was identified through RT-qPCR. **B** The level of PUM2 was assessed by Western blot analysis. **C** TTC staining detected the infarct size of brain tissues. **D** H&E staining showed the pathological histological damages of the brain tissues. **E** TUNEL assay revealed the apoptosis of neural cells. Five mice were used in each group. ***p < 0.001 relative to sham+LV-shNC group; ^#^*p* < 0.05, ^###^*p* < 0.001 relative to sham+LV-shPUM2; ^&&&^*p* < 0.001 relative to MCAO+LV-shNC

Next, PUM2 was downregulated in MCAO model to test the function of PUM2 in cerebral I/R. The inhibitory efficiency of LV-shPUM2 was confirmed in Fig. 2A. The expression level of PUM2 protein was dramatically decreased after LV-shPUM2 transfection in the MCAO model (Fig. 2B). Moreover, the increased infarcted size of the brain tissues in the MCAO group was obviously reduced by the inhibition of PUM2 (Fig. 2C). Besides, the damage of tissues in the MCAO model were also alleviated as a result of PUM2 silencing (Fig. 2D). Additionally, the apoptosis of neural cells in MCAO group was decreased by suppression of PUM2 (Fig. 2E). These results showed that PUM2 restraint alleviated MCAO-induced brain injury.

### PUM2 silencing increased the survival of neural cells exposed to OGD/R

**Fig. 3:**
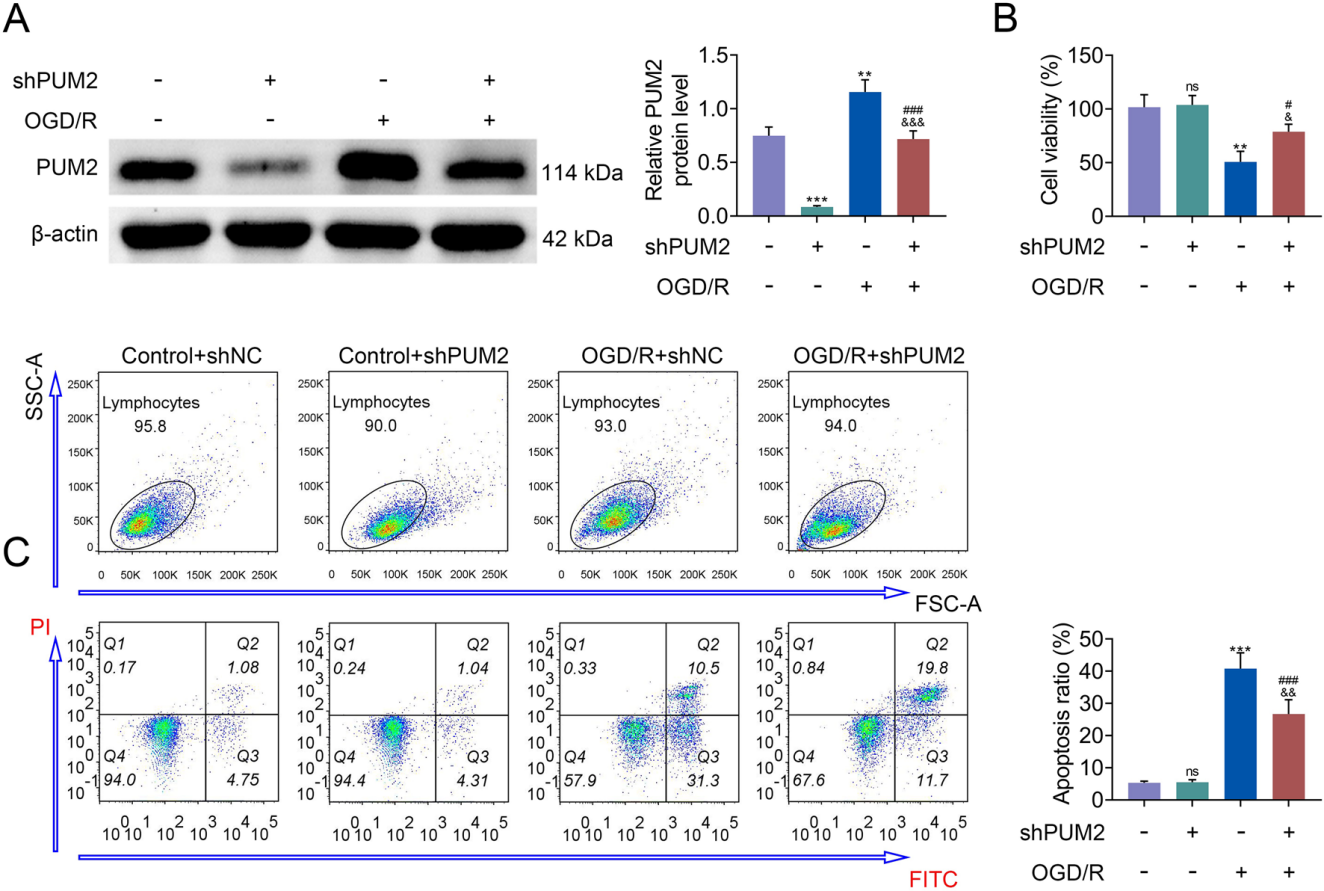
PUM2 silencing increased the survival of neural cells exposed to OGD/R. **A** PUM2 protein level was evaluated by Western blot analysis. **B** MTT assay was applied to test the viability of cortical neurons. **C** Flow cytometry analysis was employed to examine the apoptosis of cortical neurons. **p < 0.01, ***p < 0.001 relative to control+shNC group; ^#^*p* < 0.05, ^#^*p* < 0.01, ^###^*p* < 0.001 relative to control+shPUM2; ^&^*p* < 0.05, ^&&&^*p* < 0.001 relative to OGD/R+shNC

Next, the function of PUM2 was measured in cortical neurons exposed to OGD/R. The level of PUM2 was knocked down in cortical neurons, which uncovered that the protein level was prominently downregulated by the transfection of shPUM2 (Fig. 3A). The data from MTT assay unveiled that the decreased cell proliferation in OGD/R group was increased by PUM2 silencing (Fig. 3B). Meanwhile, the apoptosis of cortical neurons was increased in OGD/R group, which was reversed by PUM2 silencing (Fig. 3C). Overall, PUM2 silencing increased the survival of neural cells exposed to OGD/R.

### PUM2 mediated ferroptosis

**Fig. 4:**
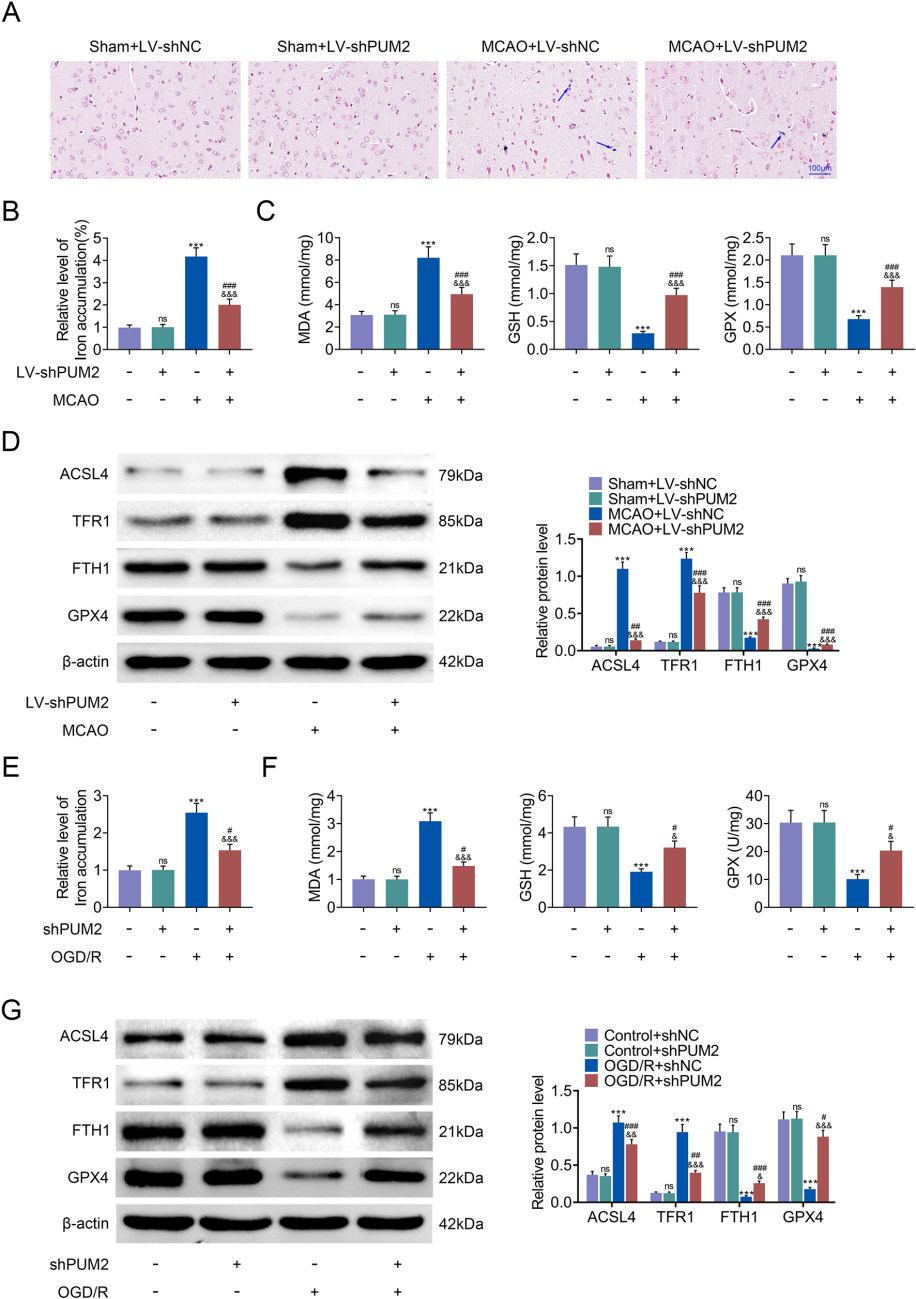
PUM2 mediated ferroptosis. **A**, **B** Prussian blue staining uncovered that the accumulation of the free iron in cells from MCAO model. **C** The concentrations of MDA, GSH and GPX in brain tissues were tested by ELISA. **D** Western blot analysis measured the protein levels of ACSL4, TFR1, FTH1 and GPX4 in MCAO model. **E** Iron detection kit showed that the accumulation of the free iron in cortical neurons from OGD/R model. **F** The concentrations of MDA, GSH and GPX in OGD/R-induced cortical neuron injury model were detected by ELISA. **G** Western blot analysis measured the protein levels of ACSL4, TFR1, FTH1 and GPX4 in OGD/R-induced cortical neuron injury model. Five mice were used in each group. **p < 0.01, ***p < 0.001 relative to sham+LV-shNC or control+shNC group; ^#^*p* < 0.05, ^#^*p* < 0.01, ^###^*p* < 0.001 relative to sham+LV-shPUM2 or control+shPUM2; ^&^*p* < 0.05, ^&&&^*p* < 0.001 relative to MCAO+LV-shNC or OGD/R+shNC

Since ferroptosis has been demonstrated to be implicated with cell death in the cerebral I/R [23], the role of PUM2 in ferroptosis in I/R was further explored. The Prussian blue staining results uncovered that the accumulation of the free iron in cells in the MCAO group was remarkably elevated compared with that in sham group, which was reversed by PUM2 silencing in MCAO group (Fig. 4A, B). Moreover, the elevated concentration of MDA and decreased concentrations of GSH and GPX in brain tissues from MCAO group were counteracted by PUM2 inhibition (Fig. 4C). Additionally, the levels of ferroptosis-related proteins (ACSL4, TFR1, FTH1 and GPX4) were detected by Western blot analysis, and results showed that the levels of ACSL4 and TFR1 were increased in the MCAO group, while the levels of FTH1 and GPX4 were decreased, but these effects were reversed by PUM2 silencing (Fig. 4D). Similarly, the increased free iron deposition in the OGD/R group was also attenuated by PUM2 knockdown (Fig. 4E). The elevated concentration of MDA and suppressed concentrations of GSH and GPX in OGD/R group were offset by PUM2 silencing (Fig. 4F). In consistent, the increased levels of ACSL4 and TFR1 and decreased levels of FTH1 and GPX4 in OGD/R group were reversed by PUM2 reduction (Fig. 4G). In a word, PUM2 mediated ferroptosis.

### PUM2 suppressed SLC7A11 via the inhibition of SIRT1

**Fig. 5:**
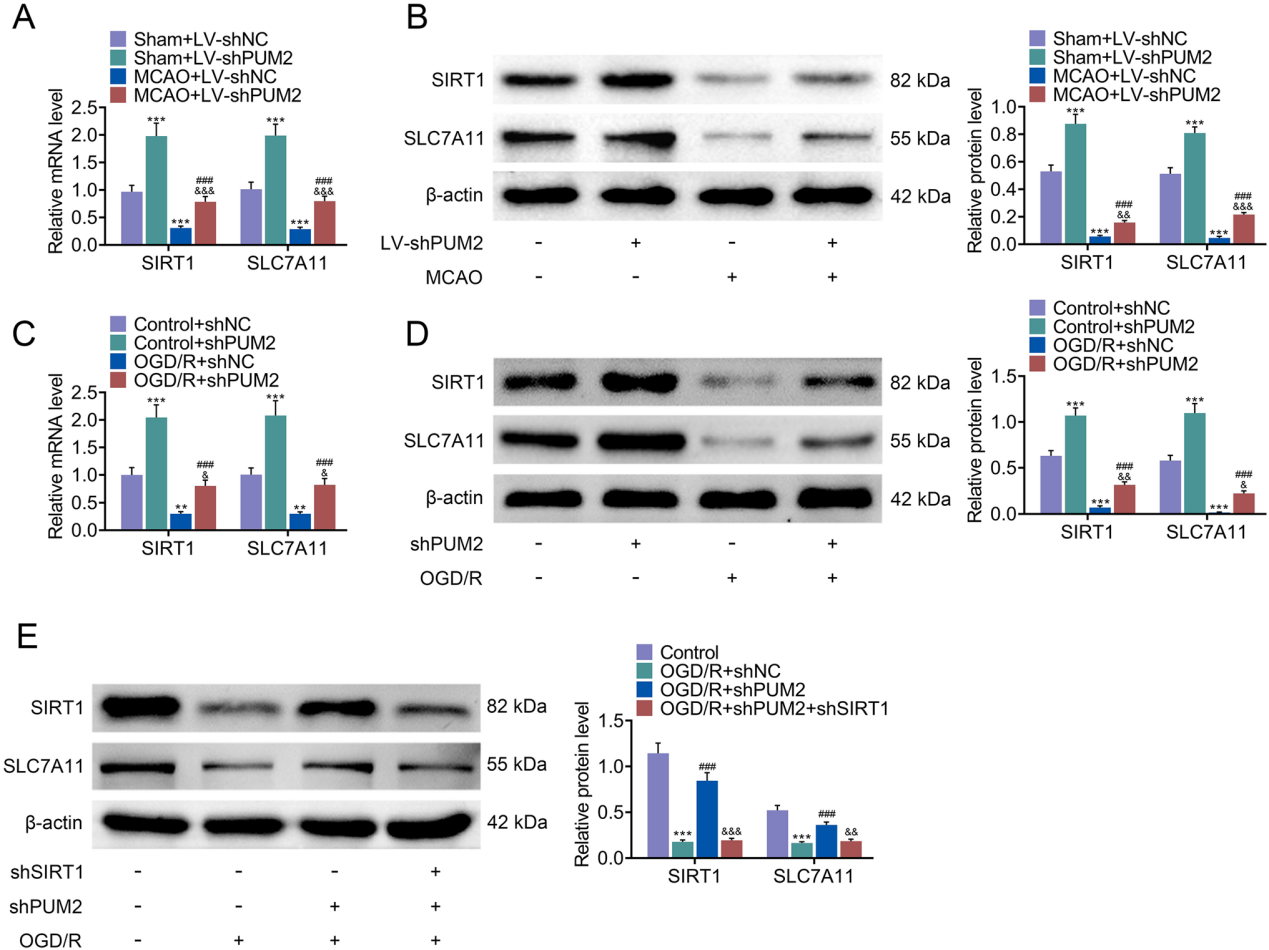
PUM2 suppressed SLC7A11 via the inhibition of SIRT1. **A**, **B** The mRNA and protein levels of SIRT1 and SLC7A11 in MCAO model were assessed by RT-qPCR and Western blot analysis. Five mice were used in each group. ***p < 0.001 relative to sham+LV-shNC; ^###^*p* < 0.001 relative to sham+LV-shPUM2; ^&&^*p* < 0.01, ^&&&^*p* < 0.001 relative to MCAO+LV-shNC. **C**, **D** The mRNA and protein levels of SIRT1 and SLC7A11 in OGD/R-induced cortical neuron injury model were evaluated through RT-qPCR and Western blot analysis. **p < 0.01, ***p < 0.001 relative to control+shNC group; ^###^*p* < 0.001 relative to control+shPUM2; ^&^*p* < 0.05, ^&&&^*p* < 0.001 relative to OGD/R+shNC. **E** The protein level of SLC7A11 in OGD/R-induced cortical neurons injury model was evaluated by Western blot analysis. ***p < 0.001 relative to control group; ^#^*p* < 0.05, ^#^*p* < 0.01 relative to OGD/R+shNC; ^&^*p* < 0.05, ^&&^*p* < 0.01 relative to OGD/R+shPUM2

Furthermore, the regulatory mechanism of PUM2 in I/R was explored. As presented in Fig. 5A and B, downregulation of PUM2 elevated the mRNA and protein expression levels of SIRT1 and SLC7A11 in the sham group. Compared with sham+LV-shNC group, the mRNA and protein levels of SIRT1 and SLC7A11 were reduced in MCAO+LV-shNC group, but these effects were inversely changed by PUM2 silencing. Consistently, the mRNA and protein levels of SIRT1 and SLC7A11 were elevated by PUM2 suppression in the control group. Compared with that in the control+shNC group, the mRNA and protein levels of SIRT1 and SLC7A11 were decreased in the OGD/R+shNC group, which was counteracted by PUM2 inhibition (Fig. 5C, D). Besides, SIRT1 restraint reversed PUM2 downregulation mediated elevation of the SLC7A11 protein level (Fig. 5E). Taken together, PUM2 suppressed SLC7A11 via the inhibition of SIRT1.

### SLC7A11 downregulation reversed PUM2 mediated neuroinflammation and brain damage

**Fig. 6:**
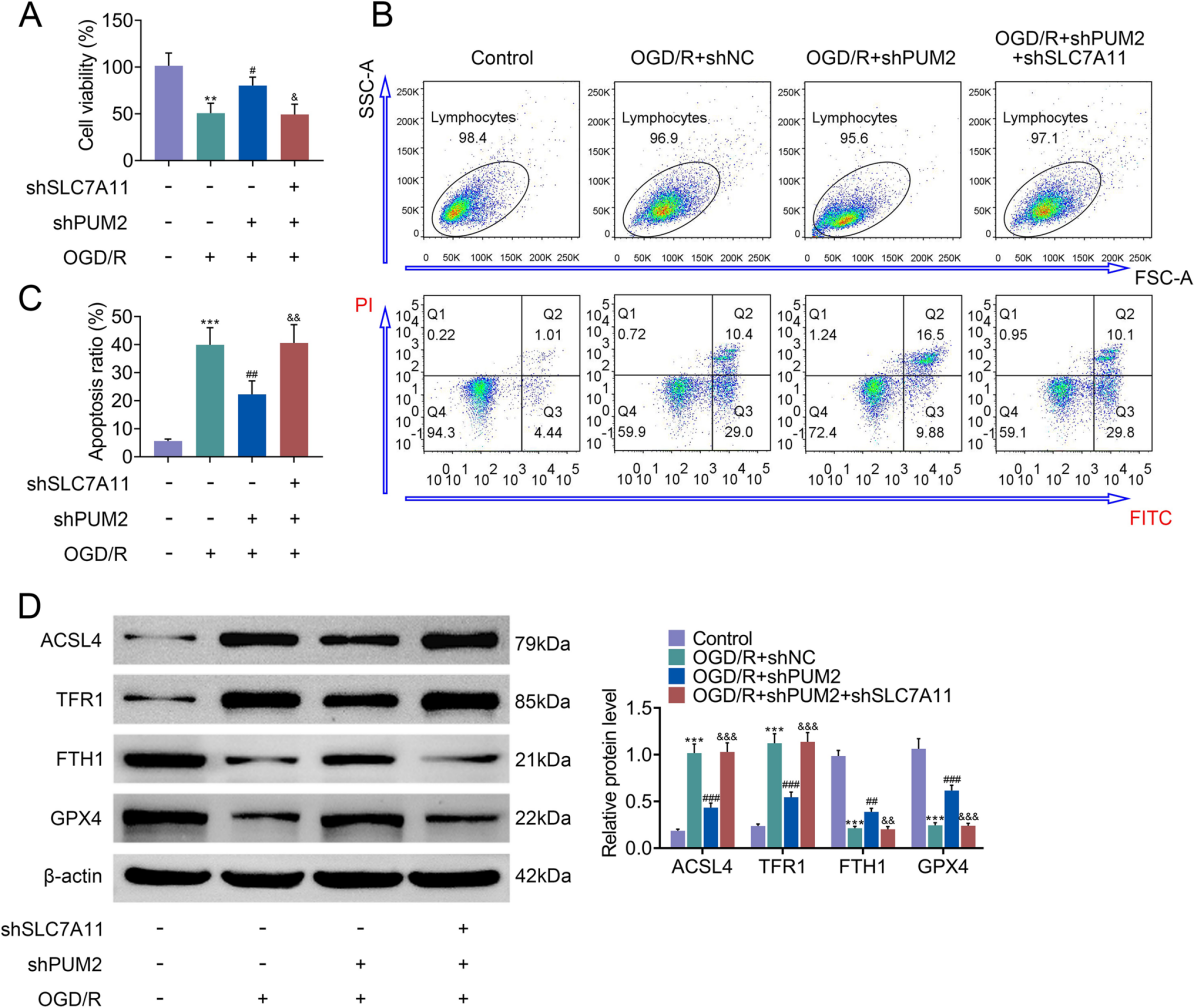
SLC7A11 downregulation reversed PUM2 mediated neuroinflammation and brain damage. **A** The viability of cortical neurons exposed to OGD/R was measured by MTT assay. **B**, **C** The apoptosis of cortical neurons exposed to OGD/R was detected via flow cytometry analysis. **D** Western blot analysis measured the protein levels of ACSL4, TFR1, FTH1 and GPX4 in cortical neurons exposed to OGD/R. *p < 0.05, **p < 0.01 relative to control group; ^#^*p* < 0.05, ^##^*p* < 0.01 relative to OGD/R+shNC; ^&^*p* < 0.05, ^&&^*p* < 0.01 relative to OGD/R+shPUM2

Subsequently, whether PUM2 modulated neuroinflammation and brain damage via mediating SLC7A11 was investigated. The data from MTT assay unveiled that decreased cell viability in OGD/R group was reversed by PUM2 depletion, but this effect was offset by SLC7A11 downregulation (Fig. 6A). In contrast, the increased apoptosis of cortical neurons in OGD/R group was decreased by PUM2 inhibition, which was counteracted by SLC7A11 suppression (Fig. 6B, C). In addition, PUM2 reduction reversed the increased levels of ACSL4 and TFR1 and decreased levels of FTH1 and GPX4 in OGD/R group, but these effects were offset by SLC7A11 silencing (Fig. 6D). Thus, SLC7A11 downregulation reversed PUM2 mediated neuroinflammation and brain damage.

## Discussion

Cerebral I/R injury occurs frequently due to the restoration of blood perfusion after cerebral ischemia and hypoxia, resulting in the damage of the brain structures and functions and leading to some serious complications [24]. Unfortunately, there are currently no effective methods for the prevention and treatment of cerebral I/R injury. Thus, it is critical to explore novel biomarkers participating in the process of cerebral I/R. Previously, mRNAs were verified to be closely related to the development of cerebral I/R. For instance, Notch2 served as a target of miR-149-5p to promote cerebral I/R [25]. NOX2 mediated the inflammatory microenvironment and reactive oxygen species production in the cerebral I/R injury [26]. Map2k6 played an important role in modulating I/R-induced neuron cell apoptosis [27]. PUM2 was validated to be involved in the development of ovarian cancer [10], acute ischemic kidney injury [11] and mature neurons [12]. Nonetheless, whether PUM2 is involved in cerebral I/R injury still needs to be elucidated. Herein, the cerebral I/R model including animal MCAO model and OGD/R-induced cortical neuron injury cell models were established. The mRNA and protein levels of PUM2 were elevated in both the brain tissues of the MCAO model and OGD/R-induced cortical neuron injury model. PUM2 silencing alleviated MCAO-induced brain injury and decreased cell death in OGD/R-induced cortical neuron injury model.

Ferroptosis, presented as iron accumulation, lipid peroxidation, and mitochondrial membrane density condensation, is a type of regulated cell deaths [28]. In previous studies, ferroptosis has been validated to be implicated with various processes of diseases, including neurological diseases. For example, lncRNA PVT1/miR-214/TFR1/TP53 axis modulated I/R progression through regulating ferroptosis [29]. Ferroptotic neuronal death was suppressed by miR-212-5p in traumatic brain injury through modulating Ptgs2 [30]. GTP cyclohydrolase I mediated microglial activation via regulating the potential targets of miRNAs through ferroptosis in neuropathic pain [31]. Whether PUM2 mediated I/R injury via ferroptosis is unclear. In our study, PUM2 aggravated the accumulation of the free iron in MCAO mice and OGD/R-induced cortical neuron injury model. In summary, PUM2 modulated cerebral I/R injury via mediating ferroptosis.

Ferroptosis is influenced by a variety of critical factors, including the vital transporter of cysteine, and solute carrier family 7 member 11 (SLC7A11), which transports extracellular cystine into the cells [32, 33]. Then, SLC7A11 is converted into glutathione (GSH), which can be used by GPX4 to reduce lipid hydroperoxides for suppressing the ferroptosis [34]. SLC7A11 has been verified to modulate ferroptosis in multiple studies [35]. For instance, EZH2-mediated SLC7A11 modulated ferroptosis in acute liver failure [36]. SLC7A11, mediated by EZH2, regulated the ferroptosis in tongue squamous cell carcinoma [37]. SLC7A11 controlled the development of lung cancer via ferroptosis [38]. In the current study, whether PUM2 aggravated the neuroinflammation and brain damage induced by I/R through SLC7A11 was investigated.

SIRT1 is frequently reported to be involved in cerebral I/R injury, and owns neuroprotective effects [17–19]. This study identified that PUM2 silencing markedly increased the levels of SIRT1 and SLC7A11. PUM2 suppressed SLC7A11 via inhibiting SIRT1. Rescue assays indicated that SLC7A11 downregulation reversed PUM2 mediated neuroinflammation and brain damage induced by I/R.

In conclusion, this study found that PUM2 aggravated the I/R-induced neuroinflammation and brain damage through the SLC7A11-dependent inhibition of ferroptosis via suppressing the SIRT1. These findings might highlight the role of PUM2 in preventing or treating cerebral I/R injury.

## Data Availability

All data generated or analyzed during this study are included in this published article.
